# Molecular Modeling and Simulation Analysis of Antimicrobial Photodynamic Therapy Potential for Control of COVID-19

**DOI:** 10.1155/2022/7089576

**Published:** 2022-05-31

**Authors:** Maryam Pourhajibagher

**Affiliations:** Dental Research Center, Dentistry Research Institute, Tehran University of Medical Sciences, Tehran, Iran

## Abstract

Severe acute respiratory syndrome coronavirus 2 (SARS-CoV-2) can enter the host cells by binding the viral surface spike glycoprotein (SG) to angiotensin-converting enzyme 2. Since antiviral photodynamic therapy (aPDT) has been described as a new method for inhibiting viral infections, it is important to evaluate whether it can be used as a photoactivated disinfectant to control COVID-19. In this *in silico* study, SARS-CoV-2-SG was selected as a novel target for curcumin as a photosensitizer during aPDT to exploit its physicochemical properties, molecular modeling, hierarchical nature of protein structure, and functional analysis using several bioinformatics tools and biological databases. The results of a detailed computational investigation revealed that SARS-CoV-2-SG is most similar to 6VXX_A, with 100% query cover and identity. The predicted structure of SARS-CoV-2-SG displayed that it is a protein with a positive charge and random coil dominates other secondary structures located outside the viral cell. The protein-protein interaction network showed that SARS-CoV-2-SG interacted with ten potential interacting partners. In addition, primary screening of binding modes through molecular docking showed that curcumin desires to bind and interact with residues of SARS-CoV-2-SG as the main site to enhance the yield of aPDT. Overall, the computer simulation reveals that SARS-CoV-2-SG can be a suitable target site for interaction with curcumin during aPDT.

## 1. Introduction

Coronavirus disease (COVID-19), known as a new form of pneumonia disease that emerged in Wuhan, Hubei province (China), in winter 2019, is caused by severe acute respiratory syndrome coronavirus 2 (SARS-CoV-2) which has confronted the world with the dire state of global emergency health [1]. SARS-CoV-2 is an enveloped virus with a positive-sense, single-stranded RNA genome with sizes ranging from 26 to 32 kilobases in length [2]. The SARS-CoV-2 genome encodes four major structural glycoproteins to produce a structurally complete viral particle which includes the nucleocapsid (N), membrane (M), envelope (E), and spike (S) proteins [3]. It has been described that SARS-CoV-2 can enter the host cells by binding the viral surface spike glycoprotein to angiotensin-converting enzyme 2 [4]. The spike glycoprotein forms homo-trimers protruding from the viral surface; especially, the spike 1 surface unit allows the attachment of the virus to cellular receptors [3, 4]. Despite extensive research conducted by scientists and researchers, there is still no effective antiviral therapy to eradicate the virus. However, reports are available on the application of antimicrobial photodynamic therapy (aPDT) to combat viral infections [5–7].

The initial clinical applications of aPDT as a potentially effective and safe treatment modality for infectious diseases were directed against superficial viral diseases. PDT is associated with the administration of a photosensitizing agent as a photosensitizer, which selectively accumulates in the target cells and activates following local irradiation with visible light.

Curcumin, 1,7-bis(4-hydroxy-3-methoxyphenyl)-1,6-heptadien-3,5-dione, as a nontoxic lipophilic molecule, can rapidly permeate host cell membrane [8]. As mentioned previously, curcumin has been proven to be effective in a wide range of activities against various viruses [9–11]. As commented on by Chen et al. [12], curcumin may display antiviral activity against influenza A virus propagation due to being a strong inhibitor of NF-кB signaling and inhibits hemagglutinin (HA) as a viral protein in the influenza A virus. In addition, curcumin significantly decreased the viral titer at subcytotoxic doses. As studies indicate, curcumin targets the early stages of virus infection, especially the attachment stage in the virus life cycle [12]. Therefore, in this *in silico* study, curcumin was considered a photosensitizer in aPDT.

Recently, several clinical trials of systemic and topical aPDT as a treatment approach and adjuvant therapy with surgery, cryotherapy, or chemotherapy have been conducted against viral infections [5, 7, 13]. Currently, there are two main clinical and medical aspects for the aPDT: (1) the treatment of local viral infections and (2) the area of blood product decontamination. Many reports have also emerged over the years of the use of aPDT as an effective methodology for sterilization of blood or blood products [13–15]. According to the literature, SARS-CoV-2 not only causes respiratory tract infections but also can be detected in blood plasma [16].

Several studies have revealed that lipid-enveloped viruses are more susceptible to inactivation using aPDT than nonenveloped viruses [17]. aPDT damages to the structure of the viral envelope can inhibit the binding of the virus to the cells. Nevertheless, it is important to identify the viral relevant molecular targets with appropriate sensitivity to aPDT. Based on the results of our previous studies [18–20], *in silico* analysis of proteins enhances our knowledge of the target site to modify the photosensitizer for increasing the efficiency of aPDT.

In the current study, we used a number of bioinformatics tools and biological databases to predict the physicochemical properties, molecular modeling, and structure validation of spike glycoprotein of SARS-CoV-2 (SARS-CoV-2-SG) as one of the important factors to virus entry into the host cells in order to evaluate the effectiveness of aPDT with curcumin as a photosensitizer.

## 2. Methods

### 2.1. Retrieval of the Sequences

The amino acid sequence of SARS-CoV-2-SG was gained from the National Center for Biological Information (NCBI; http://www.ncbi.nlm.nih.gov) with the GenBank ID of QIC53213.1 in FASTA format for computational analysis. Similar sequences to SARS-CoV-2-SG were then retrieved through the Protein Basic Local Alignment Search Tool (BLAST; https://www.ncbi.nlm.nih.gov/blast/) was conducted in Protein Data Bank (PDB; http://www.rcsb.org).

### 2.2. Hierarchical Nature of Protein Structure

#### 2.2.1. Primary Sequence Analysis

Universal Protein Resource (UniProt) (http://expasy.org) and Expasy ProtParam server (http://www.expasy.org/cgi-bin/protpraram) were used to predict the physicochemical properties and functional characterization of SARS-CoV-2-SG such as such isoelectric point (pI), molecular weight (Mw), extinction coefficient (EC-quantitative study of protein-protein and protein-ligand interactions), instability index (II-stability of proteins), aliphatic index (AI-relative volume of protein occupied by aliphatic side chains), and grand average of hydropathicity index (GRAVY-sum of all hydropathicity values of all amino acids divided by the number of residues in a sequence).

#### 2.2.2. Secondary Structure Prediction

The helices, sheets, and turns of amino acid sequences related to the secondary structure of SARS-CoV-2-SG were predicted by PSI-blast-based secondary structure PREDiction (PSIPRED; http://bioinf.cs.ucl.ac.uk/psipred/) and Garnier–Osguthorpe–Robson (GOR) IV (http://cib.cf.ocha.ac.jp/bitool/GOR/).

#### 2.2.3. Protein Three-Dimensional Model Prediction

The query sequence taken was the amino acid sequence of spike glycoprotein (severe acute respiratory syndrome coronavirus 2). So the comparative homology protein model was predicted through the SWISS-Model Workspace (https://swissmodel.expasy.org/interactive). The quality of the SARS-CoV-2-SG structure was validated by means of programs PROCHECK, ERRAT, VERIFY3D, and SAVES that are available at the structure analysis and verification server (http://nihserver.mbi.ucla.edu/SAVES/).

SARS-CoV-2-SG was analyzed as the target based on the Global Model Quality Estimation and Qualitative Model Energy Analysis (QMEAN) scores. The interaction energy per residue and confirmation of the local and overall model quality of SARS-CoV-2-SG were calculated by means of the ProSA-web server (https://prosa.services.came.sbg.ac.at/prosa.php). As well as, the signal peptide was predicted using the SignalP-5.0 server (http://www.cbs.dtu.dk/services/SignalP/).

### 2.3. Protein-Protein Interactions Network Analysis

STRING (http://string-db.org) was utilized as an approach to assess the protein-protein interactions network and rank their significance or validity as targets. Moreover, the query sequence was also analyzed to determine the family of proteins using the motif finder server (http://www.genome.jp/tools/motif/). The Protter server (http://wlab.ethz.ch/protter/) was then used to determine the interactive integration and embodiment of annotated and predicted protein sequence features together with experimental proteomic evidence.

### 2.4. Protein-Photosensitizer Docking

As previously reported, docking studies were performed to identify the preferred orientation and molecular interactions of natural compounds with targeted proteins. In this current study, the binding patterns and affinity estimations for the interaction between SARS-CoV-2-SG and curcumin were performed using molecular docking (SYBYL-X 2.1 program). The top-ranked conformation with the lowest binding energy was selected.

## 3. Results

### 3.1. Sequence Retrieval Analysis

Amino acid sequence identity analysis at the NCBI GenBank database showed that SARS-CoV-2-SG had 1255 amino acids, with an estimated molecular weight of 139125.14 Da (average mass) and 139035.85 (monoisotopic mass). The amino acid compositions of SARS-CoV-2-SG are as follows: Ala (A) 6.2%; Arg (R) 3.3%; Asn (N) 6.9%; Asp (D) 4.9%; Cys (C) 3.1%; Gln (Q) 4.9%; Glu (E) 3.8%; Gly (G) 6.4%; His (H) 1.3%; Ile (I) 6.0%; Leu (L) 8.5%; Lys (K) 4.8%; Met (M) 1.1%; Phe (F) 6.0%; Pro (P) 4.6%; Ser (S) 7.8%; Thr (T) 7.6%; Trp (W) 0.9%; Tyr (Y) 4.2%; Val (V) 7.6%; and Pyl (O) and Sec (U) 0%.

From the BLAST results, PDB ID: 6VXX_A was found with maximum similarity. The total score for protein alignment between SARS-CoV-2-SG and 6VXX_A with 1281 amino acids was 2657, with 100% and 100% query cover and identity, respectively.

### 3.2. Physicochemical Characterization

Physicochemical properties have a key role in the characterization of a specific protein. Basic information from the initial structure showed that SARS-CoV-2-SG had 103 positively charged residues (Arg + Lys) and 110 negatively charged residues (Asp + Glu). The aliphatic index was 84.67 which suggested that the protein is thermostable. Its theoretical isoelectric point (PI), extinction coefficient at 280 nm measured in water, and the grand average of hydropathicity (GRAVY) values were 6.24, 148960 M^−1^cm^−1^, and −0.079, respectively. The lower range of GRAVY indicated that a better interaction was established between protein and water.

The estimated half-life of SARS-CoV-2-SG was 30 h (mammalian reticulocytes, *in vitro*), >20 h (yeast, *in vivo*), and >10 h (*Escherichia coli*, *in vivo*). As a result, it is a stable protein that can be a therapeutic target for aPDT.

### 3.3. Secondary Structure Prediction

**Figure 1:**
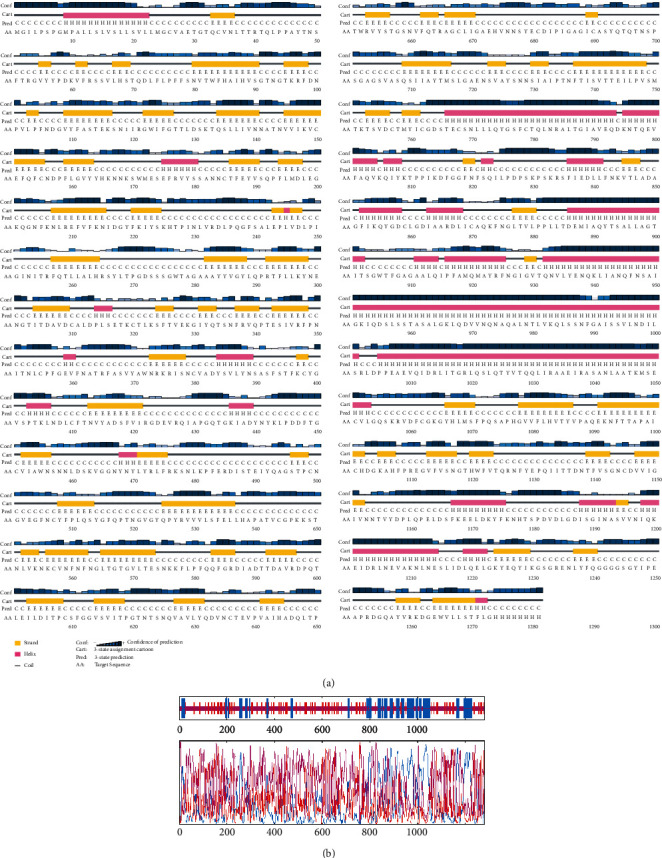
Secondary structure prediction of SARS-CoV-2-SG using (a) PSIPRED and (b) GOR IV.

Secondary structure prediction using PSIPRED and GOR IV showed the location and spatial arrangement of each 6VXX_A amino acid separately (Figures 1(a) and 1(b)). As the figure shows, the random coil (Cc: 57.69%, 739 residues) has dominance over other secondary structures, whereas the alpha helices (Hh: 23.33%, 286 residues) and the extended strand (Ee: 19.98%, 256 residues) were the least frequent.

### 3.4. Protein Three-Dimensional Modeling and Functional Analysis

**Figure 2:**
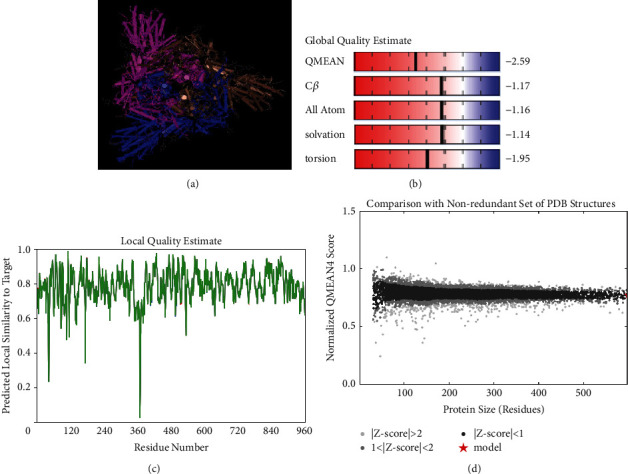
Bioinformatics analysis and computer simulation molecular modeling of SARS-CoV-2-SG. (a) Three-dimensional structures, (b) QMEAN value, (c) local model quality overall model quality, and (d) comparison with a nonredundant set of SARS-CoV-2-SG structure.

A three-dimensional structure of SARS-CoV-2-SG is shown in Figure 2(a). The selected template sequence was 6VXX_A with a query sequence of 100% and a resolution of 2.80 angstroms (Ǻ). The oligo state of the predicted protein model was a homo-trimer. The QMEAN and global score were −2.59 and 0.76 ± 0.05, respectively (Figures 2(b)–2(d)).

**Figure 3:**
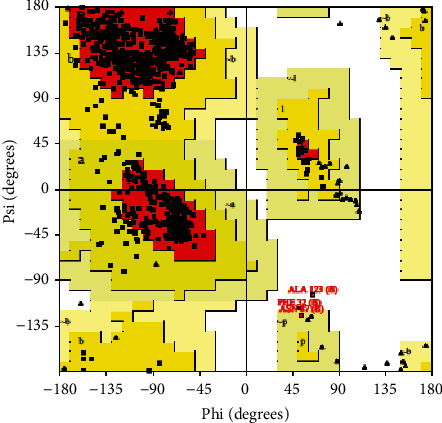
Ramachandran plot of SARS-CoV-2-SG by PROCHECK server.

Based on an analysis of 118 structures of resolution of at least 2.0 Ǻ and an R-factor no greater than 20%, a good quality model would be expected to have over 90% in the most favored regions. According to the Ramachandran plot analysis, 90.0% (2277/2529) of residues fall in the most favored region [A, B, L]; among them, 243 residues (9.6%) were present in the additional allowed region [a, b, l, p]; and 3 (0.1%) and 6 (0.2%) residues in generously allowed [∼a,∼b,∼l,∼p] and disallowed regions [xx], respectively (Figure 3). The numbers of end-residues (excl. Gly and Pro), glycine residues, and proline residues were 69, 174, and 144, respectively.

**Figure 4:**
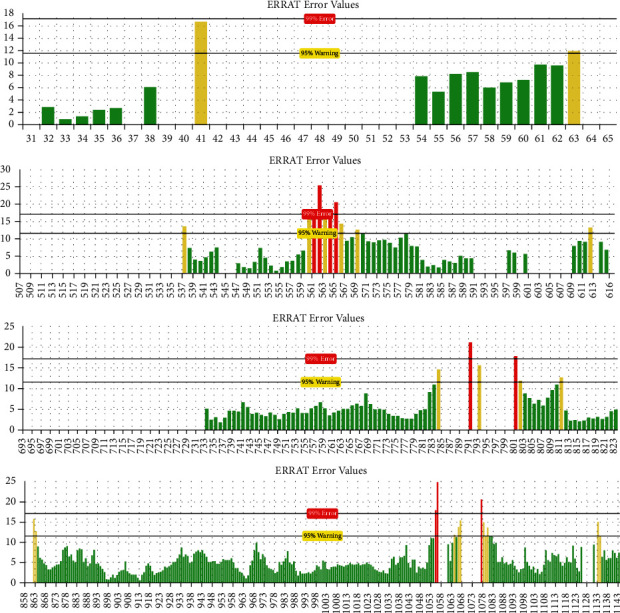
Evaluation of protein model of SARS-CoV-2-SG by ERRAT.

The mean overall quality factor of the predicted model by ERRAT was 92.20, which implicated that any bonded atomic interactions of the generated model were not within the normal range (Figure 4).

**Figure 5:**
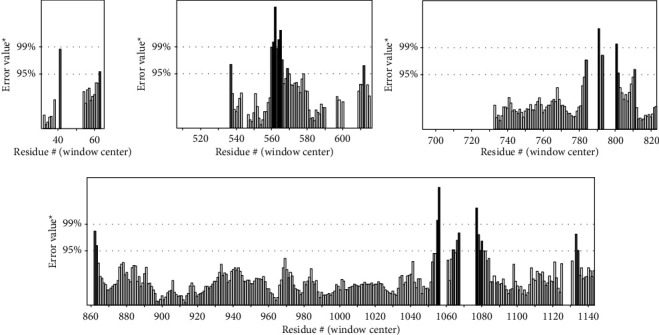
Evaluation of protein model of SARS-CoV-2-SG by SAVES.

As shown in Figure 5, the error axis, two lines are drawn to indicate the confidence with which it is possible to reject regions that exceed that error value. Also, expressed as the percentage of the protein for which the calculated error value falls below the 95% rejection limit. Good high-resolution structures generally produce values around 95% or higher. For lower resolutions (2.5 to 3A) the average overall quality factor is around 91%. The results of SAVES in the current study show that the overall quality factor is 95.05.

**Figure 6:**
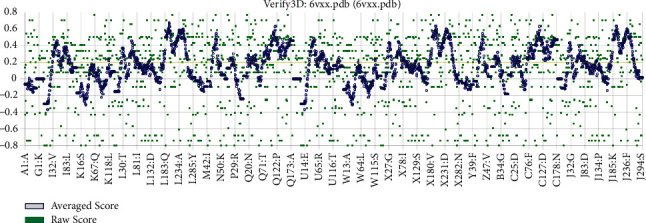
Evaluation of protein model of SARS-CoV-2-SG by VERIFY3D.

VERIFY3D a table computed from the atomic coordinates of the structure was used to score the compatibility of the three-dimensional structure model with each amino acid sequence. The finding in Figure 6 verified the correctness of a protein model by the compatibility of its three-dimensional profile with its sequence.

**Figure 7:**
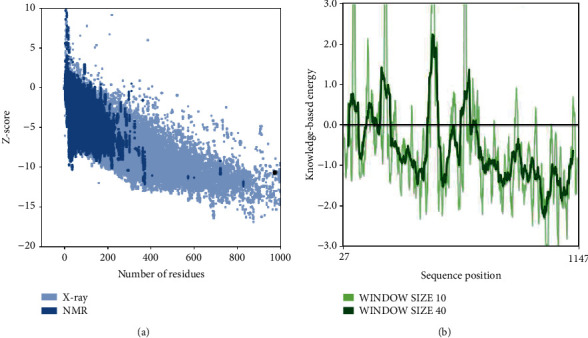
ProSA output for SARS-CoV-2-SG. (a) Overall model quality with ProsaWeb server and (b) local model quality with ProsaWeb server.

The ProSA-web results of local and overall model quality of the predicted model exhibited that the energy profile of the SARS-CoV-2-SG is consistent with the reliable conformation with the Z-Score of −10.65 (Figure 7). Hence, results gained from PROCHECK, ERRAT, VERIFY3D, SAVES, and ProSA indicate that the modeled structure of SARS-CoV-2-SG is reasonable and reliable as a therapeutic target for aPDT.

### 3.5. Protein-Protein Interactions Network Analysis

**Figure 8:**
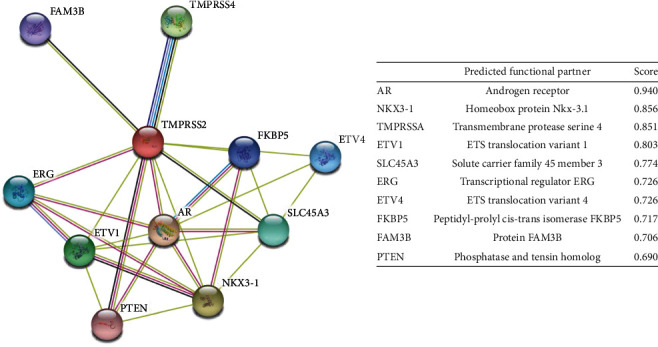
Protein-protein interaction map of SARS-CoV-2-SG by STRING.

Functional analysis by STRING revealed ten potential interacting partners of transmembrane protease serine 2 (TMPRSS2) in the protein interaction network (Figure 8). Serine protease proteolytically cleaves and activates the viral SP. As shown in Figure 6, the protein-protein interaction network, their length, and their type of relationship with TMPRSS2 are displayed.

The closest interacting proteins having the shortest node were found to be AR (an androgen receptor) and FKBP5 (as a peptidyl-prolyl cis-trans isomerase FKBP5), while the distant interacting proteins were found to be PTEN (phosphatase and tensin homolog) and ETV4 (ETS translocation variant 4).

**Figure 9:**
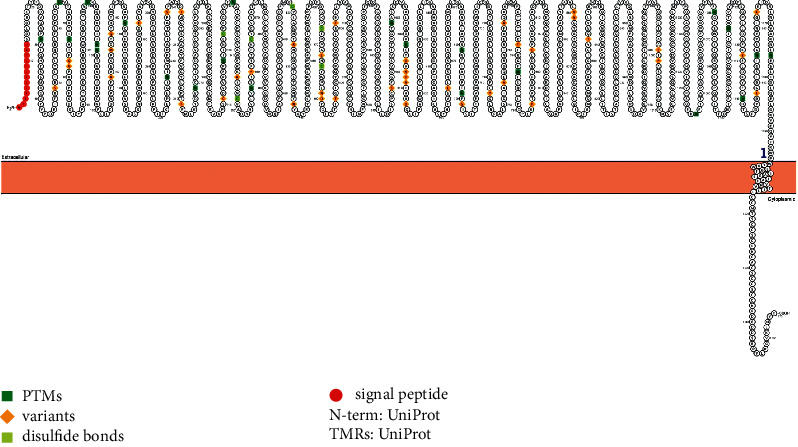
Identification of signal peptide in SARS-CoV-2-SG by PROTTER.

The result of the signal peptide identification in SARS-CoV-2-SG is shown in Figure 9. The signal peptide is located at amino acids 1–13 (MFUFLLFLTLTSG). As well, the positions of posttranslational modification (PTM), variants, and disulfide bonds are shown in Figure 9.

**Figure 10:**
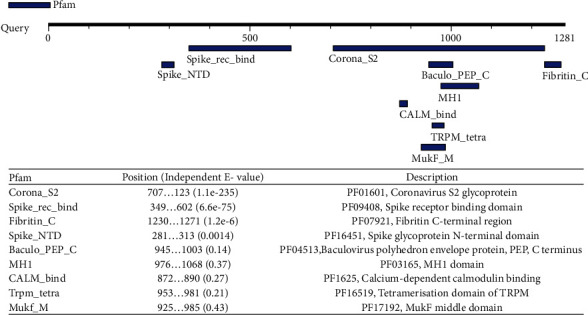
Result of motif finder in SARS-CoV-2-SG.

In addition, nine functional motifs were also detected from the functional study in the case of SARS-CoV-2-SG (Figure 10).

### 3.6. Molecular Docking Study

**Figure 11:**
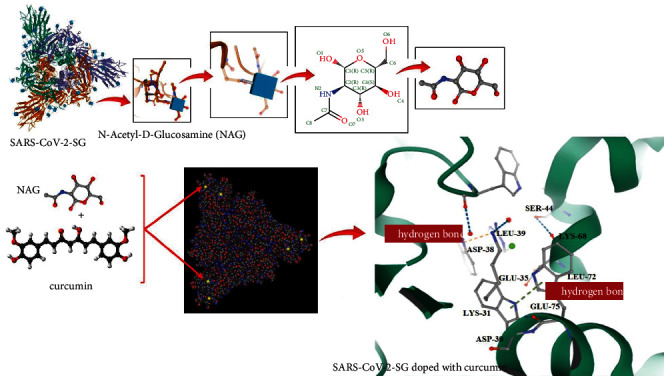
Docking analysis between ligands of the modeled SARS-CoV-2-SG doped with curcumin.

The molecular interaction of curcumin with SARS-CoV-2-SG was identified by performing a molecular docking study (Figure 11). The best docking orientation was chosen based on the lowest energy value (DG_bind_) and negative. Docking analysis showed a strong bond between curcumin and SARS-CoV-2-SG with the lowest energy value (−6.2 kcal/mol). Key hydrogen bond interactions play a significant role for all ligands involving ASP-30, LYS-31, GLU-35, ASP-38, LEU-39, SER-44, LYS-68, LEU-72, and GLU-75 along with other residues. Given the results from docking analysis, our study provides evidence that this identified molecule interacting with SARS-CoV-2-SG specifically would prove to be a potential target for aPDT.

## 4. Discussion

The World Health Organization (WHO) has already declared COVID-19 infection as a global pandemic problem and that there is no antiviral treatment against SARS-CoV-2 [16]. The effect of aPDT as adjunct therapy on viral infections such as the Middle East respiratory syndrome (MERS), and influenza caused by Middle East respiratory syndrome coronavirus (MERS-CoV) and orthomyxoviruses, respectively, can be generalized to COVID-19 due to the similarity of viral pathogenesis to cause primarily mild to severe respiratory infections. In humans, these viruses can be detected with higher viral load and longer duration in the respiratory tract and have been detected also in faces, serum, urine, and blood samples, causing millions of infections each year [21–27].

A recent study by Jin et al. [25] conducted aPDT with methylene blue could be effective against SARS-CoV-2 in plasma without any side effects. Previously, it has been proven that methylene blue photochemical technology not only inactivates *in vitro* the lipid-enveloped viruses but also can be applied in clinical trial treatment without damage to other components in the plasma [26, 27]. According to the Jin et al. results, 1, 2, and 4 *μ*M of methylene blue photochemical therapy under the wavelength of 630 nm light for 2 minutes could completely inactivate the virus and the viral titer of SARS-CoV-2 decreased to 4.5 log_10_ median tissue culture infectious dose (TCID50)/mL [25].

As commented on by Eickmann et al. [26, 27], methylene blue plus visible light at light doses as low as 30 J/cm^2^, or 25% of the standard full light dose of 120 J/cm^2^ is capable of reducing SARS-CoV and MERS-CoV by more than 3.1 and 3.3 log_10_ TCID_50_/mL in plasma, respectively. Therefore, according to previous studies, aPDT has been suggested for the control of COVID-19. It is confirmed that not only the type and concentration of photosensitizer as well as parameters of the light fluency play a role in aPDT, but also the subcellular localization of the photosensitizer in the target site of host cells plays the main role in the cell death mechanism during aPDT [28].

Although there is no evidence specifically investigating the effect of aPDT on viral lipids and/ or proteins, there are investigations about the effect of ROS on viral lipids [29–32]. Since there are viral lipids in the envelope of COVID-19, it should probably be sensitive to the effects of aPDT. The disparate effectiveness of aPDT can be due to variations in the target sites of the photosensitizer. On the other hand, it is unknown which structure of the virus can bind to the photosensitizer. In this study, we introduced the SG as a target site against SARS-CoV-2 using molecular modeling and simulation analysis through numerous data banks. As we know, prediction of the three-dimensional structure of a protein by bioinformatics research because of the nonavailability of the crystal structures is a highly challenging aspect to confirm the data obtained from the NMR or X-ray crystallographic based methods [33].

In concordance with the results obtained, SARS-CoV-2-SG was a homo-trimer, thermostable protein, having an average molecular weight of 139125.14 Da with nine functional motifs. As reported [34, 35], molecular docking is very useful and reasonably reliable for the prediction of putative binding modes and affinities of macromolecules (receptors) and small molecules (ligands). The more negative docking score reveals stronger binding between ligand and receptor. The results of the molecular docking study by Sohilait et al. [34] revealed the binding orientations of curcumin analogues in the active sites of cyclooxygenase-2 towards the design of potent inhibitors with anti-inflammatory activities. In another study, Hobani et al. [35] studied the interaction of curcumin with the enzyme dihydrofolate reductase (DHFR) by molecular docking. They reported curcumin can be considered an appropriate compound in the development of new inhibitors of DHFR, which is a potential target of anticancer drugs.

The findings of this study revealed that curcumin and SARS-CoV-2 had a strong bond with a binding energy value of −6.2 kcal/mol. During aPDT, curcumin binds into the SARS-CoV-2-SG and can penetrate into cells following light irradiation. Eventually, aPDT at a wavelength of 450 nm can collapse the structure of the viral cells through the generation of ROS and prevent the attachment of viruses to the host cell surface.

This *in silico* study might be a valuable contribution in the field of bioinformatics research in the control of COVID-19 and may help other researchers to get an idea about the protein structure, its physicochemical properties, and protein-protein interaction to conduct extensive examinations on the design and manufacture of drugs and vaccines against COVID-19.

## 5. Conclusion

Selected protein structural properties were visualized to select an appropriate photosensitizer and a specific wavelength of light to show the effectiveness and interpretability of the aPDT against COVID-19. Taken together, computer simulation reveals that SARS-CoV-2-SG can be a suitable target site for interaction with curcumin. Also, it suggests the aPDT can be effective against COVID-19 by targeting the spike glycoprotein of SARS-CoV-2 as well as the virus entry step. Further studies to assess the current outcome are needed to achieve better performance of aPDT against COVID-19.

## Data Availability

All datasets supporting the conclusions of this article are included within the article.
